# Supplementary material for: Response Mechanisms of Zelkova schneideriana Leaves to Varying Levels of Calcium Stress
Source: Int J Mol Sci. 2024 Aug 27;25(17):9293. doi: 10.3390/ijms25179293 (PMC11394862; doi:10.3390/ijms25179293)
Supplement: Supplementary file 1 [file ijms-25-09293-s001.zip › ijms-3126029-supplementary.pdf]

Table S1: Statistical table of sequencing data.

Sample	Raw reads	Clean reads	Q20(%)	Q30(%)	GC content (%)
CK_1	48470300	48128890	97.15	91.64	45.59
CK_2	47730380	47398794	97.11	91.52	45.19
CK_3	46932532	46594686	97.16	91.69	45.53
Ca20_1	48579810	48220124	97.08	91.46	45.53
Ca 20_2	44263806	43897850	96.97	91.17	45.51
Ca 20_3	45467748	45089110	97.27	92	45.39
Ca 100_1	50053924	49679690	97.19	91.75	45.63
Ca 100_2	46706510	46379936	97.01	91.29	45.93
Ca 100_3	41769010	41423240	97.02	91.29	45.71

Table S2: Evaluation of assembly results.

Type	Unigene	Transcript
Total number	52916	97470
Largest length (bp)	14645	14645
Smallest length (bp)	201	201
Average length (bp)	932.38	1010.85
N50 length (bp)	1672	1673
E90N50 length (bp)	2377	2138
Fragment mapped percent(%)	68.17	81.128
GC percent (%)	40.2	40.25
TransRate score	0.34225	0.40386

Table S3: Primers for real-time fluorescence quantitative PCR.

Gene ID	Gene annotation	Primer Sequence
TRINITY_DN1632_c1_g3	*CNGC*	F: CGGATACAGAAAAAACAG<br>R: AGATATTACATGCCACCT
TRINITY_DN5772_c0_g2	*CPDK*	F: GGCTCCAATAGCAACAAC<br>R: ATCCTCTTCACCGCAAC
TRINITY_DN5487_c0_g1	*KCS*	F: TGGAGTTATGTGAGGGTGG<br>R: CCGAAATCTGTGGCTTTTA
TRINITY_DN6768_c0_g1	*DFR*	F: TGGAGTTATGTGAGGGTGG<br>R: CCGAAATCTGTGGCTTTTA
TRINITY_DN21964_c0_g1	*LAR*	F: TCCCACCACTGAAAAAAG<br>R: AAACTCTGACGGATAAAACC
TRINITY_DN3038_c0_g1	*PAL*	F: GCAGCGATTGGGTTATG<br>R: CCAGAGTAGCCTTGGAGAA
TRINITY_DN3889_c0_g3	*PHOSPHO2*	F: CAATCTTCACGGGCACCA<br>R: GGCATCACAAAGTCTCCC
TRINITY_DN8458_c0_g1	*petE*	F: AGACTTACGCTGTGACCTTG<br>R: AACAGTAACTTTTCCCACCA
TRINITY_DN7046_c0_g1	*psbY*	F: CGGGAGAGACTTACGCTGTG<br>R: TGGGGTTGGTGATTTTGGT
Reference gene	*UBC*	F: TCCTTCTATTGGGTTTG<br>R: CTGAGATGGGATGGTAA
